# Comparison of the effectiveness of **Satureja khuzestanica** and clotrimazole vaginal creams for the treatment of vulvovaginal candidiasis

**DOI:** 10.25122/jml-2020-0014

**Published:** 2021

**Authors:** Shirin Jaldani, Mahnaz Fatahinia, Elham Maraghi, Eskandar Moghimipour, Mojgan Javadnoori

**Affiliations:** 1. Department of Midwifery, School of Nursing and Midwifery, Ahvaz Jundishapur University of Medical Sciences, Ahvaz, Iran; 2. Infectious and Tropical Diseases Research Center, Health Research Institute, Ahvaz Jundishapur University of Medical Sciences, Ahvaz, Iran; 3. Department of Medical Mycology, School of Medicine, Ahvaz Jundishapur University of Medical Sciences, Ahvaz, Iran; 4. Medicinal Plant Research Center, Ahvaz Jundishapur University of Medical Sciences, Ahvaz, Iran; 5. Department of Biostatistics and Epidemiology, Faculty of Public Health, Ahvaz Jundishapur University of Medical Sciences, Ahvaz, Iran

**Keywords:** Satureja Khuzestanica, clotrimazole, candidal vulvovaginitis

## Abstract

Candidal vaginitis has a relatively high prevalence, and its resistance to treatment is on the rise. Considering the complications of chemical drugs, the use of herbal medicines has now been favored due to the lack of changes in the normal vaginal flora. The aim of this study was to compare the effectiveness of *Satureja khuzestanica* and clotrimazole vaginal creams for the treatment of candidal vulvovaginitis. A randomized clinical trial was conducted on 84 reproductive-aged women in the city of Ahvaz, Iran. Individuals were randomly divided into two treatment groups: 1% *Satureja khuzestanica* vaginal creams (n=42) and 1% clotrimazole vaginal cream (n=42) who used a one-full applicator daily for one week. About 4–7 days after the end of treatment, a clinical examination and laboratory re-tests were performed to determine the level of treatment. The data were analyzed using the Mann-Whitney U, t-test and Chi-square tests, with SPSS version 22. After the treatment, no significant difference was observed between the two groups in terms of vaginal discharge (p = 0.32), vaginal itching (p = 0.26), dysuria (p = 0.99) and dyspareunia (p = 0.60). Moreover, the results of culture (p = 0.62) and smear (p = 0.58) were not statistically significant in the two groups. Also, there was no significant difference between the two groups in terms of complete recovery after the treatment (p = 0.35). *Satureja khuzestanica* seems to have the same effect as clotrimazole in improving the symptoms of vaginal candidiasis, the negative results of culture and smear, as well as complete treatment.

## Introduction

Genital tract infection is one of the most common causes of women’s visits to treatment centers. Candidal vaginitis is the second most common cause of vaginitis in women and is diagnosed in more than 40% of women in health centers [1].

Women develop vaginal candidiasis at least once (75%) or twice (50%) in their lives, and 5% of most women develop it more than four times a year [2, 3]. *Candida albicans* is responsible for 85–90% of vaginal candidiasis and has more ability to stick to vaginal epithelial cells than other *Candida species* [4]. Other types of *Candida*, such as *Candida glabrata* and *Candida tropicalis*, which also cause vaginal candidiasis, are usually resistant to treatment [5]. Their prevalence has been reported between 25–45% [6, 7].

Symptoms of vaginal candidiasis include genital itching, thick and cheesy discharge, vaginal irritation, dysuria, and dyspareunia [8]. Diagnosis is usually based on clinical symptoms and direct microscopic examination. Direct microscopic examination is a rapid clinical method that may identify etiologic factors; however, the result of vaginal discharge culture seems definitive as a diagnosis [9]. The most common culture medium for diagnosing *Candida* is the suburase-dextrose medium, and in some cases, it is possible to use the CHROMagar medium [10].

Clotrimazole is the most accessible and widely used treatment for vaginal candidiasis [11]. 

Dysuria, depression due to systemic absorption, itching, dermatitis, irritation, and itching in the partner’s genital organ are also side effects of topical antifungal drugs [12]. On the other hand, the preventive and therapeutic use of antifungal medicines has caused an increased drug resistance to these drugs, followed by the increased side effects of medications [13]. Therefore, given the side effects reported for clotrimazole as an approved treatment for candidal vulvovaginitis and other chemical drugs recommended for the treatment of vaginal candidiasis, the use of substances that have an antifungal effect and at the same time have fewer side effects seems necessary. Today, the use of herbal-based drugs that are more compatible with the body, especially with no changes in the normal vaginal flora, is considered [14].

*Satureja khuzestanica* is a plant in the mint family. The essential oil of this plant is prepared in the glandular trichome of the epiphyte that contains high amounts of carvacrol drug combinations. Carvacrol is the most important compound of essential oil of *Satureja* species that has the ability to affect the cytoplasmic membrane, electron transport chain, metabolic activity, gene synthesis, inhibits protein synthesis, and also has antimicrobial and antimicrobial properties [15]. Satureja is a traditional herbal medicine acting as a stimulus, anti-flatulence, expectorant, stomach tonic, and a sexual enhancer with anti-inflammatory effects [16]. The anti-diabetic, anti-parasitic, and anti-oxidant effects of *Satureja khuzestanica* have been confirmed [17].

A recent study has shown that *Satureja khuzestanica* has strong anti-candida effects on vaginal discharge in a laboratory environment by inhibiting germ tube formation [18]. Another study showed that Satureja bachtiarica vaginal cream with clotrimazole is not only similar to clotrimazole but also has synergistic effects on improving the symptoms of vaginal candidiasis [19]. According to laboratory studies conducted in the field of antifungal effects of *Satureja khuzestanica*, no study was conducted on vaginal cream and its local effects on vaginal candidiasis. The aim of this study was to compare the effect of *Satureja khuzestanica* and clotrimazole vaginal creams on the treatment of vulvovaginal candidiasis.

## Material and Methods

This is a double-blind clinical trial performed on 84 reproductive-aged women within the reproductive age in the Eastern Health Center of Ahvaz. 

We included married women with an age range between 18 and 45 years, who had symptoms indicative of vaginal candidiasis, a positive direct observation test, positive vaginal discharge culture, known medical conditions and sensitivity to Satureja and clotrimazole. The exclusion criteria were pregnant and lactating women, postmenopausal women, those taking contraceptive pills, taking broad-spectrum antibiotics over the past two weeks, using oral or vaginal-related vaginitis drugs in the last two weeks, those who do not come for smear and culture test after the treatment, not taking the complete medication, having trichomonas vaginitis and a history of repeated vaginal candidiasis. 

Using previous studies [20] and taking into account the power of 90%, α = 0.01, p1=45.5% and p2 = 85.5%, the sample size was calculated using the following formula. By including a 20% loss, a total of 84 subjects were assigned to two treatment groups (Satureja cream n=42) and (clotrimazole cream n=42). 


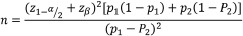


*Satureja* vaginal cream was prepared in the Faculty of Pharmacy for the use of 42 people. Accordingly, the oil phase of the cream, including Eucerin, stearic acid, and mineral oil, was weighed as per calculation and put in a flask at 70°C on a Bain Marie to melt. The aqueous phase, containing benzyl alcohol monophasic and diphasic DSP, citric acid and water, was calculated and placed in another flask on a Bain Marie to melt uniformly at the phase temperature. Then, the aqueous phase was added to the oil phase and was slowly stirred. When the temperature of 35°C was reached, the essential oil was added and stirred until the ambient temperature was reached in order to get a uniform cream. Clotrimazole cream was also discharged to tubes similar to those of *Satureja* vaginal cream and the tubes were called A and B, respectively. 

Sampling was done using convenient sampling, and the method of assigning the drug and placebo to the patients was based on permutated block randomization with block size 4 (using a permutated randomization table). A randomized list was provided by a statistician. The drugs used in this study were placed in a sealed envelope pocket, according to the corresponding codes by a person not involved in the study, and then assigned to each patient enrolled in the study. The medications were identical in terms of appearance, such as packaging and color, and thus, the researcher and patient were not aware of the type of drug.

**Figure 1.**
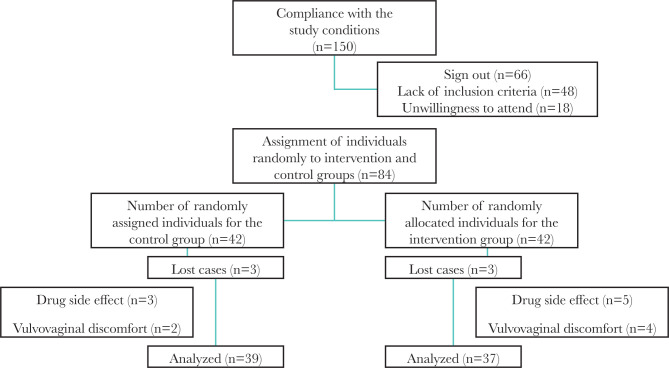
Study diagram.

Vaginal candidiasis was confirmed by symptoms, clinical examinations, and laboratory tests. To become aware of the personal information questionnaire, the symptom and observation checklist was used. In this checklist, vaginal candidiasis symptoms such as vaginal itching and discharge, dyspareunia, and dysuria were classified into four groups: none, mild, moderate, and severe. Patients had a vaginal examination to assess the clinical manifestations (amount and type of vaginal discharge). The vaginal acidity was measured by the pH-gauge strip, and a pH greater than 4.5, suggestive of the presence of other microorganisms, led to the exclusion of the subject from the study. In patients with positive vaginal diagnosis, samples of vaginal discharge were taken for culture and smear. To perform a culture, samples were collected using sterile swabs from the vaginal walls and then cultured on a CHROMagar medium linearly. The plates were transferred to the laboratory for storage at 37°C and cultured for 24 to 48 hours. If the number of grown colonies reached ten or more in each culture medium, candidal vaginitis was confirmed. The *Candida* species were also determined based on the color of the colony created on the CHROMagar medium, in which the green colony was representative of *Candida albicans*; blue colony denoted *Candida tropicalis*, pink-purple colony indicated *Candida glabrata* and pale purple colony represented *Candida krusei*. After confirming the *candidal vaginitis* through culture or stained smear, the subjects were contacted to visit the health center in order to receive the drug and start the treatment. They were advised to use a one full applicator daily for one week and refrain from taking other vaginal drugs and antibiotics, as well as having intercourse without condoms. Health advise and training were given to patients for the correct use of the drug. Patients were requested to go to the health center seven days after completing the course of treatment. Upon the patient’s visit, a vaginal examination was performed again; samples were taken for direct observation, the pH of the vagina was measured, necessary laboratory tests were performed, and then the form (after intervention) was completed. It should be noted that in the event of disapproval of candidal vaginitis in the first stage or lack of treatment, the patient was treated according to the specialist’s recommendation or was referred to midwifery or women’s clinic for treatment. Five patients from the *Satureja* treatment group and three from the clotrimazole treatment group were excluded. Finally, 37 and 39 patients were treated in the *Satureja khuzestanica* and clotrimazole groups, respectively (Figure 1).

The Chi-square test was used to examine the relationship between qualitative variables, and the independent t-test or non-parametric equivalent (Mann-Whitney U test) was used to compare quantitative variables between the two groups. The significance level of the above tests is considered below 0.01. Data were analyzed using the SPSS software, version 22.

## Results

The mean ages of the patients in the *Satureja* and clotrimazole treatment groups were 31.57 and 31.12 years, respectively. There was no significant statistical difference between the two groups according to the independent t-test (p = 0.78). Most of the subjects were overweight (25.47±2.86) in both groups in terms of body mass index (BMI), but there was no statistically significant difference between them (p = 0.11). Moreover, there was no statistically significant difference between occupation, education, economic status, contraceptive method, and health level between the two groups (Table 1).

**Table 1:** Comparison of frequency and percentage of qualitative demographic and fertility characteristics in terms of the study groups.

**Variables**	**Control group %**	**Intervention group %**	**P-value**
**Occupation**			0.99
Housewife	35 (83.3%)	35 (83.3%)	
Employed	7 (16.7%)	7 (16.7%)	
**Education**			0.353
Primary	8 (19.0%)	5 (11.9%)	
High school	12 (28.6%)	7 (16.7%)	
Diploma	12 (28.6%)	17 (40.5%)	
Academic	10 (23.8%)	13 (30.0%)	
**Economic status**			0.459
Good	10 (23.8%)	14 (33.3%)	
Average	21 (50.0%)	21 (50.0%)	
Poor	11 (26.2%)	7 (16.7%)	
**The method of contraception**			0.974
Intrauterine device	8 (19.0%)	9 (21.4%)	
Condom	7 (16.7%)	8 (19.0%)	
Natural	22 (52.4%)	20 (47.6%)	
Shot	5 (11.9%)	5 (11.9%)	

Table 2 shows the main treatment indicators during the study, including vaginal discharge, itching, dysuria, and dyspareunia. Each group showed a significant difference in the level of vaginal discharge, itching, irritation, dysuria, and dyspareunia before and after the intervention, which means that both drugs were effective in treating symptoms (Table 2). However, the Chi-square test showed no significant difference between the two groups in terms of the improvement of symptoms before and after the intervention (Table 2). 

**Table 2:** Evaluation of the main treatment indicators including vaginal discharge, itching, irritation, dysuria and dyspareunia in the study groups.

**Variables**		**Intervention group (n=42)**	**Control group (n=42)**	**P-value**
		**%**	**%**	
**Discharge** **(before treatment)**	**None**	0 (0.0)%	0 (0.0)%	0.549
	**Mild**	9 (21.4%)	13 (31.0)	
	**Moderate**	14 (33.3%)	14 (33.3%)	
	**Severe**	19 (45.2%)	15 (35.7%)	
**Discharge** **(After treatment)**	**None**	5 (13.5%)	1 (2.6)	0.329
	**Mild**	19 (51.4%)	25 (64.1%)	
	**Moderate**	8 (21.6%)	8 (20.5%)	
	**Severe**	5 (13.5%)	5 (12.8%)	
**P-value**		<0.0001	<0.0001	
**Itching (before treatment)**	**None**	0 (0.0)%	1 (2.4%)	0.132
	**Mild**	9 (21.4%)	11 (26.2%)	
	**Moderate**	17 (40.5%)	23 (54.8%)	
	**Severe**	16 (38.1%)	7 (16.7%)	
**Itching (after treatment)**	**None**	20 (54.1%)	16 (41.0%)	0.262
	**Mild**	14 (37.8%)	14 (35.9%)	
	**Moderate**	3 (8.1%)	7 (17.9%)	
	**Severe**	0 (0.0)%	2 (5.1%)	
**P-value**		<0.0001	<0.0001	
**Irritation (before treatment)**	**None**	14 (33.3%)	23 (54.8%)	0.134
	**Mild**	14 (33.3%)	10 (23.8%)	
	**Moderate**	9 (21.4%)	8 (19.0%)	
	**Severe**	5 (11.9%)	1 (2.4%)	
**Irritation (after treatment)**	**None**	29 (78.4%)	31 (79.5%)	0.993
	**Mild**	5 (13.5%)	5 (12.8%)	
	**Moderate**	3 (8.1%)	3 (7.7%)	
	**Severe**	0 (0.0)%	0 (0.0)%	
**P-value**		<0.0001	<0.006	
**Dyspareunia (before treatment)**	**None**	24 (57.1%)	30 (71.4%)	0.022
	**Mild**	1 (2.4%)	5 (11.9%)	
	**Moderate**	13 (31.0%)	3 (7.1%)	
	**Severe**	4 (9.5%)	4 (9.5%)	
**Dyspareunia (after treatment)**	**None**	28 (75.7%)	30 (76.9%)	0.601
	**Mild**	5 (13.5%)	7 (17.9%)	
	**Moderate**	4 (10.8%)	2 (5.1%)	
	**Severe**	0 (0.0)%	0 (0.0)%	
**P-value**		<0.006	<0.044	

Using Fisher’s exact test, no statistically significant difference was observed between the two groups in terms of cultures and stained smears (Table 3).

**Table 3:** Results of culture and stained smear in the two study groups.

**Variables**		**Intervention group (n=42)**	**Control group (n=42)**	**P-value**
		**%**	**%**	
**Vaginal stained smear before treatment**	**Positive**	36 (85.7%)	32 (76.2%)	0.405
	**Negative**	6 (14.3%)	10 (23.8%)	
**Vaginal stained smear after treatment**	**Positive**	7 (18.9%)	10 (25.6%)	0.586
	**Negative**	30 (81.1%)	29 (74.4%)	
**P-value**		<0.0001	<0.0001	
**Vaginal culture before treatment**	**Positive**	42 (100.0%)	42 (100.0%)	-
	**Negative**	0 (0.0%)	0 (0.0%)	
**Vaginal culture after treatment**	**Positive**	13 (35.1%)	11 (28.2%)	0.623
	**Negative**	24 (64.9%)	28 (71.8%)	
**P-value**		<0.0001	<0.0001	

Several patients in the intervention group complained about vaginal irritation. Most patients of the control and intervention groups (45.9%) mentioned average satisfaction with their medication for treatment. Using the Chi-square test, there was no significant difference between the two groups’ satisfaction (p = 0.437).

Using Fisher’s exact test, there was no significant difference between the two drugs in terms of a complete treatment of candidal vulvovaginitis, and the two drugs had an equal effect on the complete recovery of vaginal candidiasis (p = 0.335). 

## Discussion

The results of this study showed that *Satureja khuzestanica* had a favorable therapeutic effect on candidal vaginitis by reducing the symptoms. A comparison between the two treatment groups of *Satureja khuzestanica* and clotrimazole vaginal creams showed no statistically significant difference between them.

The present study did not find a statistically significant difference between the two groups regarding vaginal discharge before and after the treatment. Therefore, it can be concluded that *Satureja khuzestanica* and clotrimazole have a similar effect in improving vaginal discharge. In a comparison study of the effect of clotrimazole with *Satureja bakhtiarica* and clotrimazole vaginal creams on the treatment of candidal vaginitis, it was shown that the vaginal discharge level was significantly lower in the combination of clotrimazole with *Satureja bakhtiarica* than clotrimazole alone, which is not consistent with the result of this study [19].

The results of this study showed that there was no statistically significant difference between the two groups before and after the treatment of vaginal itching. Therefore, it can be concluded that Satureja and clotrimazole have a similar effect in improving vaginal itching.

There was no statistically significant difference between the two groups before and after the treatment of dysuria, so we suggest that Satureja and clotrimazole have a similar effect in improving dysuria. The result of a study that investigated the effect of yogurt and honey versus clotrimazole vaginal creams in the treatment of candidal vaginitis is consistent with this study [21]. In another study, the dysuria rate was significantly lower in the combined clotrimazole and Satureja group than in the clotrimazole group [19]. The results of this study are consistent with the results of our study. 

There was no statistically significant difference between the two groups before and after the treatment of dyspareunia. In a study comparing the effect of thyme, garlic, and clotrimazole vaginal creams, there was no significant difference between the three groups after treatment [22]. Also, in another study, there was no significant difference between painful intercourse in the clotrimazole and the Satureja treatment group compared to the clotrimazole group [19]. The results of these studies are consistent with the results of the present study. 

There was no significant difference between the two groups in terms of smear and culture. This means that both treatments showed an almost equal effect on the number of negative cultures and smears after the treatment.

In a study comparing the effect of yogurt and honey vs. clotrimazole vaginal creams, no statistically significant difference was found between the two groups after the treatment [21]. The results of these studies are consistent with the result of the present study.

A study that investigated the anti-candida effects of *Satureja khuzestanica* essential oil on isolated samples from women with candidal vaginitis showed that in the disk diffusion method, the diameter of the inhibition zone increased due to the *Satureja khuzestanica* dosage. Accordingly, the diameter of the inhibition zone in *Satureja khuzestanica* (1.5 ml/disc) essential oil treatment was greater than ketoconazole (10 μg/disc) and clotrimazole (10 μg/disc). However, the diameter of the inhibition zone of *Satureja khuzestanica* (1 ml/disc) essential oil treatment was higher than amphotericin 50 mg and smaller than ketoconazole (10 μg/disc) and clotrimazole (10 μg/disc) [18]. Therefore, it can be concluded that with an increase in the concentration of *Satureja khuzestanica* essential oil, its anti-candida effects will increase.

In a study that investigated the effects of essential oils and extracts of 50 Iranian herbs on the standard strain of *Candida albicans in vitro*, it was shown that the anti-candida effects of Satureja were very strong (inhibitory zone diameter was greater than 40 mm) and outperformed amphotericin B, Ketoconazole and Nystatin, results that are consistent with the present study [23].

The limitations of the present study are the likelihood that the symptoms are not accurately reported by the research units. Differences in the immune system and physiology of individuals are another factor. The other limitations of the study were the lack of long-term follow-up; also, the effect of Satureja khuzestanic on recurrent vulvovaginal candidiasis should be investigated in future studies.

## Conclusion

Considering the presented results, it can be stated that *Satureja khuzestanica* could almost have the same results as clotrimazole in improving candidal vaginitis symptoms and the positive outcome of culture and smear tests (the negative test results). Since the cost of using herbal medicines is lower than chemical drugs and the *Satureja khuzestanica* herb is a native plant, we consider that the use of the *Satureja khuzestanica* vaginal cream is more cost-effective compared to the usual chemical drugs.

## Acknowledgments

### Funding

The authors would like to thank the research deputy of Ahvaz Jundishapur University of Medical Sciences for funding this study (grant number: RHPRC-9612).

### Ethical approval

The approval for this study was obtained from the Ethics Committee of Ahwaz Jundishapur University of Medical Sciences (Approval ID: IR.AJUMS.REC.1396.878. The protocol was registered at the Clinical Trial Registration Center (IRCT20180202038591N1).

### Consent to participate

Informed written consent was collected from the participants after the study objectives and confidentiality of patients’ profiles were explained.

### Conflict of interest

The authors declare that there is no conflict of interest.
