# Correction to: Comparison of the effectiveness of Satureja khuzestanica and clotrimazole vaginal creams for the treatment of vulvovaginal candidiasis

**DOI:** 10.25122/jml-2021-1007

**Published:** 2021

**Authors:** Shirin Jaldani, Mahnaz Fatahinia, Eskandar Moghimipour, Elham Maraghi, Mojgan Javadnoori

**Affiliations:** 1. Department of Midwifery, School of Nursing and Midwifery, Ahvaz Jundishapur University of Medical Sciences, Ahvaz, Iran; 2. Infectious and Tropical Diseases Research Center, Health Research Institute, Ahvaz Jundishapur University of Medical Sciences, Ahvaz, Iran; 3. Department of Medical Mycology, School of Medicine, Ahvaz Jundishapur University of Medical Sciences, Ahvaz, Iran; 4. Medicinal Plant Research Center, Ahvaz Jundishapur University of Medical Sciences, Ahvaz, Iran; 5. Department of Biostatistics and Epidemiology, Faculty of Public Health, Ahvaz Jundishapur University of Medical Sciences, Ahvaz, Iran; 6. Department of Midwifery, School of Nursing and Midwifery, Ahvaz Jundishapur University of Medical Sciences, Ahvaz, Iran

## Abstract

This corrects the article on p. 111 in vol. 14, PMID: 33767795.

## Correction

In the original publication [1], authorship order and the affiliation of author Mojgan Javadnoor were incorrect. The original article is now corrected. We wish to apologise for any misunderstanding or inconvenience caused.
